# An Innovative Approach to Evaluate the Morphological Patterns of Soft Palate in Oral Submucous Fibrosis Patients: A Digital Cephalometric Study

**DOI:** 10.1155/2016/5428581

**Published:** 2016-03-13

**Authors:** Chintamaneni Raja Lakshmi, Dharmavaram Ayesha Thabusum, Sujana Mulk Bhavana

**Affiliations:** Department of Oral Medicine and Radiology, Drs. Sudha & Nageswara Rao Siddhartha Institute of Dental Sciences, Gannavaram Mandal, Krishna District, Andhra Pradesh 521286, India

## Abstract

Oral submucous fibrosis (OSMF) is a chronic insidious disease affecting mucosa and submucosa of oral cavity and soft palate. The present study aimed to evaluate the morphology of soft palate in normal individuals and OSMF patients using lateral cephalometry and to compare and correlate these variants of soft palate with different stages of OSMF. 100 subjects were included in the study, who were divided into two groups. Group I included 50 subjects with clinical diagnosis of OSMF and Group II included 50 normal subjects (control group). Using digital lateral cephalometry, velar length and width were measured and soft palatal patterns were categorized based on You et al.'s classification. Leaf and rat-tail patterns of soft palate were predominant in control group, whereas butt and crook shaped variants were more in study group. Anteroposterior (A-P) length of soft palate was significantly greater in stage I OSMF, while superoinferior (S-I) width was greater in stage III OSMF. Interestingly, a negative correlation was observed in staging of OSMF and A-P dimensions. As the staging of OSMF advances, the A-P length of soft palate decreases, but S-I width increases.

## 1. Introduction

The soft palate is the posterior fibrovascular part of the palate that is attached to the posterior edge of the hard palate [1]. It participates in most of the oral functions like speech, swallowing, and respiration [2]. Although continuous research on dimensional analysis of soft palate and its surrounding structures has been made, still there is a gap in the identification of soft palate morphological patterns and configuration. Soft palate plays a very crucial role in velopharyngeal closure, that is, approximation of soft palate with pharyngeal walls. This sphincteric mechanism separates nasal and oral cavity during speech and deglutition [3]. In this context, the study of these soft palate patterns like shape, length, and width offers a clue to evaluate any risk factors for velopharyngeal incompetence. Various authors conducted studies to assess the dimensional changes of soft palate with increasing age and changes in cleft lip and palate and in patients with sleep apnea using cephalometry [4, 5] whereas studies regarding OSMF were very limited.

Oral submucous fibrosis is a chronic progressive disease affecting any part of the oral cavity and sometimes the pharynx. It is always associated with juxtaepithelial inflammatory reaction followed by fibroelastic change of the lamina propria with epithelial atrophy. The clinical manifestations include stomatitis, limited mouth opening, difficulty in speech and swallowing, and even hearing loss [6]. Oral submucous fibrosis is one such pathology that could alter the dimensions of soft tissue components like oral mucosa and soft palate; in this aspect, the present study aimed to evaluate the morphological patterns of soft palate in both normal and oral submucous fibrosis individuals. According to the literature search, to the best of our knowledge, only two studies have been reported to date.

## 2. Materials and Methods

All the procedures conducted in the present study were in compliance with the declaration of Helsinki (2000) and the study protocol was approved by the institutional ethical committee of Drs. Sudha & Nageswara Rao Siddhartha Institute of Dental Sciences, Gannavaram, India. A total of 100 subjects with age range of 20–70 years were included in this study. The subjects were randomly selected from the outpatient Department of Oral Medicine and Radiology. They were divided into two groups: Group 1 (study group) comprising 50 subjects who were clinically diagnosed with oral submucous fibrosis and Group 2 (control group) requiring lateral cephalograms for a diagnostic purpose.

Exclusion criteria were as follows: patients with history of systemic diseases, syndromes, fractures of head and neck, and cleft palate were excluded from the study.

Clinical diagnosis and staging of oral submucous fibrosis were done based on classification suggested by Nagesh and Bailoor [7].

Characteristics of stage I, early OSMF, included the following: mild blanching, no restriction in mouth opening (normal distance between central incisor tips: males, 35 to 45 mm; females, 30 to 42 mm), and no restriction in tongue protrusion. Characteristics of stage II, moderate OSMF, included the following: moderate to severe blanching, reduced mouth opening by 33%, demonstrably reduced cheek flexibility also, burning sensation also in the absence of stimuli, palpable bands felt, lymphadenopathy either unilateral or bilateral, and demonstrable anemia on hematological examination. Characteristics of stage III, severe OSMF, included the following: very severe burning sensation. The patient was unable to carry out day-to-day work, with more than 66% reduction in the mouth opening, cheek flexibility, and tongue protrusion. The tongue may appear to be fixed. Ulcerative lesions may appear on the cheek, thick palpable bands and lymphadenopathy bilaterally evident.

All lateral cephalograms were taken using digital radiographic machine (Kodak 8000 digital panoramic and cephalometric machine, Italy). A tube potential of 82 kV, a tube current of 10 mA, and an exposure time of 500 ms were used to optimize the contrast of the digital images. The digital radiographs were processed and viewed by using Kodak dental imaging software, version 6.12. Lateral cephalometric analysis was performed to evaluate the different morphological patterns of soft palate by taking the radiographs in standardized position.

All the radiographs were observed and categorized into six types according to the soft palate morphology and were evaluated by two oral and maxillofacial radiologists. The anteroposterior (A-P) length was evaluated by measuring the linear distance from the posterior nasal spine to the tip of the uvula of the resting palate. The superoinferior (S-I) width was measured at the thickest section of the velum. Different patterns of soft palate were recorded, based on classification by You et al. [1], as follows: type 1 (leaf shape), type 2 (rat-tail shape), type 3 (butt-like shape), type 4 (straight line shape), type 5 (S-shape), and type 6 (crook appearance of the soft palate).

### 2.1. Statistical Analysis

All statistical procedures were performed using Statistical Package for the Social Sciences (SPSS) version 16. The significance of the differences between the means and the distribution of various patterns of soft palate were evaluated using Mann-Whitney *U* test and Kruskal Wallis test. Correlation between the considered parameters was performed using Spearman rank correlation test. *p* value < 0.05 was set to be statistically significant.

## 3. Results

**Figure 1:**
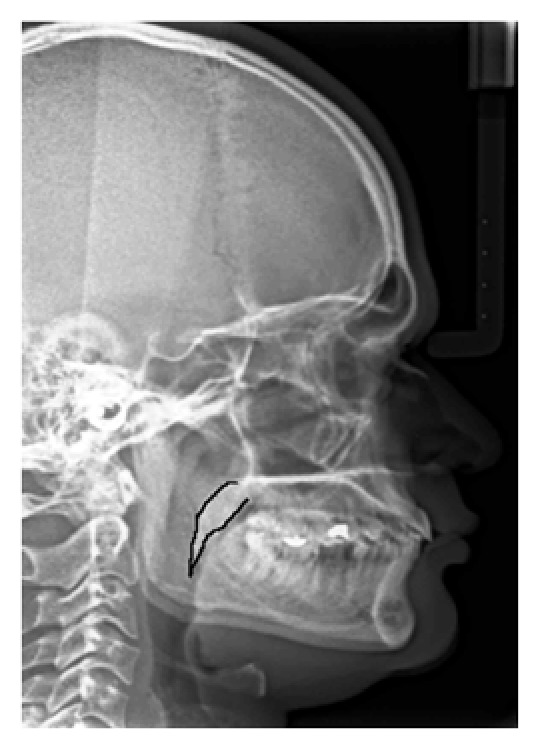
Lateral cephalogram with type 1 (leaf shaped) pattern of soft palate.

**Figure 2:**
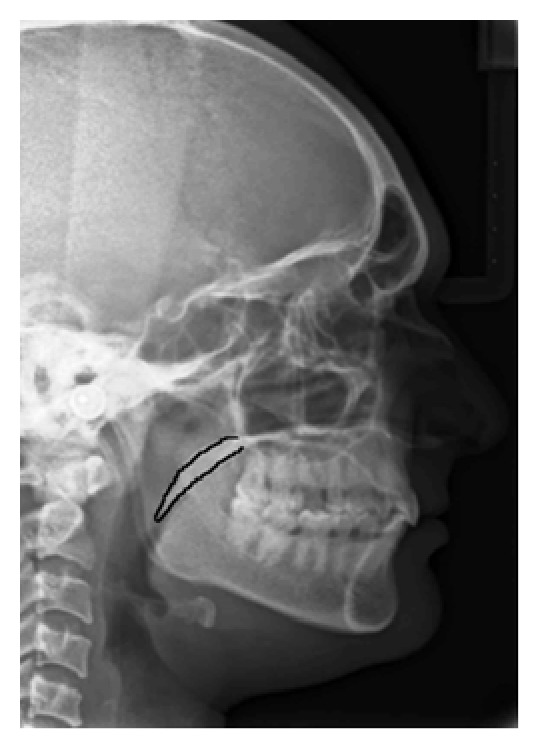
Rat-tail shape seen on lateral cephalogram.

**Figure 3:**
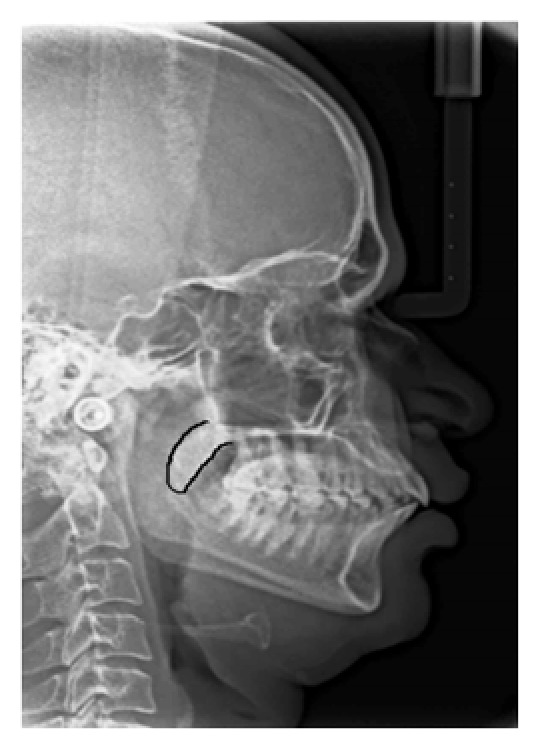
Lateral cephalogram depicting type 3 (butt shape) pattern of soft palate.

**Figure 4:**
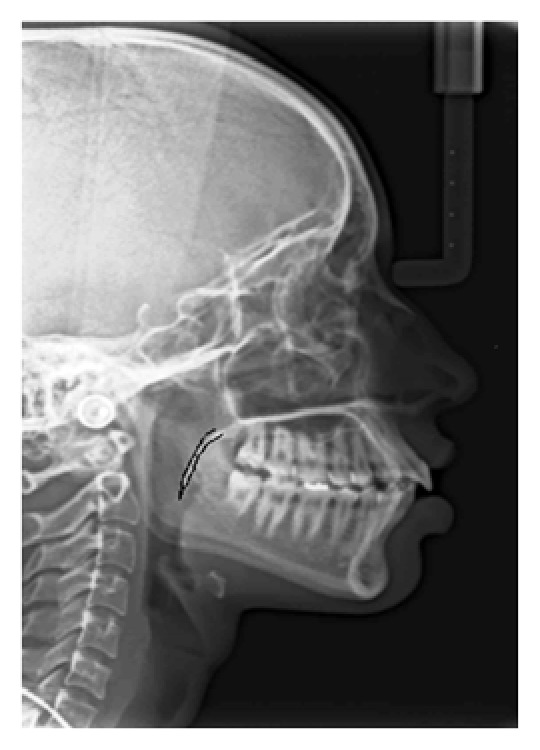
Lateral cephalogram showing type 4 (straight line) pattern of soft palate.

**Figure 5:**
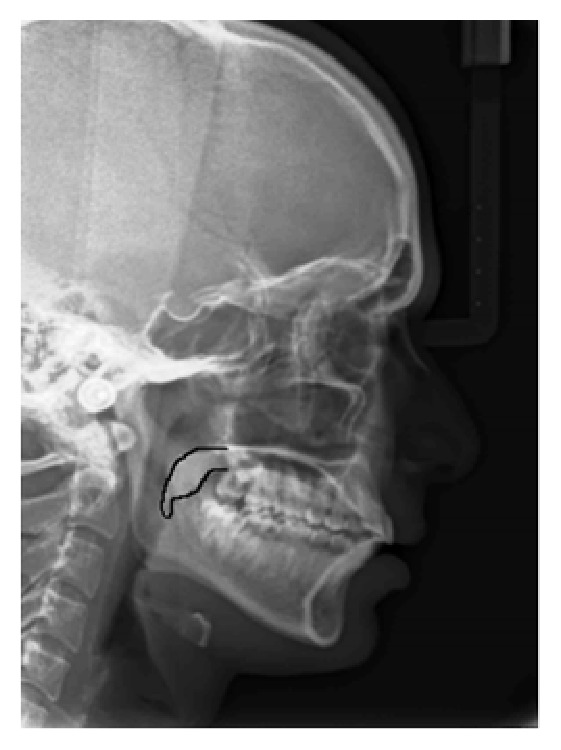
Crook shape seen on lateral cephalogram.

In the present study, the mean age of the subjects was 39 years. On the basis of the radiographic appearances in the lateral cephalograms, the soft palate patterns were categorized as type 1 (“leaf shape”), which was lanceolate, indicating that the middle portion of the soft palate elevated to both the naso- and the oro-side (Figure 1); type 2 (when the soft palate showed that the anterior portion was inflated and the free margin had an obvious coarctation, the radiographic appearance was described as having a “rat-tail shape”) (Figure 2); type 3 (“butt-like” soft palate showed a shorter and fatter velum appearance, and the width had almost no distinct difference from the anterior portion to the free margin) (Figure 3); type 4 (which indicated that the image of the soft palate presented a “straight line shape”) (Figure 4); type 5 (the distorted soft palate presented the S-shape); type 6 (which revealed a “crook” appearance of the soft palate, in which the posterior portion of the soft palate crooks anterosuperiorly) (Figure 5) [1]. The highest percentage of rat-tail shaped soft palate was present in control group. Similarly, a high percentage of butt-like and crooked shaped soft palates were present in the study group. Kruskal Wallis test was used for comparing the mean values of A-P length and S-I width in both groups according to type-wise distribution, showing significant difference (*p* < 0.05) in both A-P length and S-I width in type 1 pattern. In type 2 and 3 soft palate patterns, a difference in dimensions was significant only with A-P length and in type 6 with regard to S-I width (*p* < 0.05). No significant difference was observed (*p* < 0.05) between study and control group with respect to S-I width in types 2, 3, and 4 and A-P length in types 4 and 6 (*p* > 0.05). The highest A-P length was observed in leaf shaped soft palate, while the highest S-I width was observed in butt-like soft palate in both groups (Table 1).

Mann-Whitney *U* test was used to measure the A-P length and S-I width of soft palate showing mean value of A-P length as 32.09 in study group and 35.29 in control group. Mean value of S-I width was 10.19 and 9.23 in study and control groups, respectively, with statistically significant *p* value of 0.001. This indicates that A-P length was significantly greater in the control group, while S-I width was significantly greater in the study group. This specifies that the soft palate becomes short and stout/bulky in OSMF (Table 2).

Type-wise distribution of soft palate patterns according to staging of OSMF in the study group showed 79.16% of cases with type 1 and 55.56% of cases with type 2 soft palate pattern in stage II OSMF. 66.66% of type 3 and the highest percentage of cases showing type 6 soft palate variant were seen in stage III OSMF. One in each of stage I and II OSMF showed type 4 variant (Table 3).

The mean length and width of soft palate patterns according to stagewise distribution of OSMF revealed that the A-P length of soft palate is significantly greater in stage I than in stage II. The S-I width of stage III palate is significantly greater than of stage I and stage II (Table 4).

Interestingly, a negative correlation was observed in staging of OSMF and A-P dimensions. That is, as the staging increases, the A-P dimension decreases, but the S-I dimension increases along with staging (Table 5).

## 4. Discussion

The assessment of the soft tissue elements like soft palate and surrounding structures cephalometry is a comparatively economical method [8]. The dimensional analysis of the soft palate and its surrounding structures, especially the velar length and width, which has been overlooked in the past, is reasonably responsible for the different dimensions of the soft palate [9]. To diagnose varied pathologies of soft palate like neoplastic, neurologic, and inflammatory conditions, radiological investigations were greatly helpful. Morphological variants of soft palate play a very crucial role especially in conditions like cleft palate and obstructive sleep apnea [10]. Velopharyngeal insufficiency and hypernasal speech are still the topic of debate in surgically successful but functionally compromised cases owing to varied morphologies of soft palate and surrounding structures. Henceforth, proper assessment of soft tissue morphology is mandatory to gain better results.

Oral submucous fibrosis is a chronic insidious disease of the oral cavity affecting mucosa and submucosa of soft palate, anterior faucial pillars, buccal mucosa, tongue, and lips [11, 12]. Gaining meticulous knowledge regarding changes in soft palate morphology due to OSMF will be helpful for proper diagnosis and successful structural and functional outcome. According to literature search, there were only limited studies regarding this aspect; hence, the present study was performed to evaluate morphological changes of soft palate in OSMF patients and comparison was done with normal individuals.

In our study group, 52.2% had leaf shape soft palate which was the most common variant, with straight line variant being the least common type. The S-shape soft palate was not observed in both study and control groups. These findings were consistent with the study conducted by Shankar et al. [13]. In the control group, rat-tail shaped soft palate was predominantly seen, and straight line and crook shaped patterns were the least common. Our results were similar to a study conducted by Praveen et al. [14]. But according to You et al. [1], Kumar and Gopal [15], and Verma et al. [16], leaf shaped soft palate was described as the most common variant and crook shape as the least common variant.

In the current study, stage II OSMF was predominantly seen in 29 cases, in which type 1 pattern was seen in 19 cases (leaf shape), type 2 (rat-tail shape) in 5 cases, type 3 (butt-like shape) in 4 cases, and type 4 (straight line) in 1 case. Stage III OSMF was observed in 14 cases, in which type 1 was shown in 3 cases (leaf shape), type 3 (butt-like shape) in 6 cases, and type 6 (crook shape) in 5 cases. Seven cases were seen in stage I OSMF, among which type 1 (leaf shape) was seen in 2 cases, type 2 (rat-tail shape) in 4 cases, and type 4 (straight line) in 1 case. Based on the above findings, soft palate shows morphological changes with the progression of the disease, that is, long narrow type getting transformed into short thick pattern. These changes were attributed to the fibrosis of the mucosal covering of soft palate and uvula. These findings were in accordance with Shankar et al. and Mohan et al. [13, 17].

The mean length of type 1 (leaf shape) soft palate in the present study was 34 mm whereas for normal individuals it was 36.82 mm. The S-I width in the study group was 10.14 mm and in normal individuals it was 9.73 mm. There is significant reduction in length of the soft palate in the study group when compared with control group. This type of soft palate was seen in 7 cases of stage I, 19 cases of stage II, and 3 cases of stage III OSMF.

Type 2 (rat-tail) soft palate was noted in 4 cases of stage I OSMF and 5 cases of stage II OSMF. The mean A-P length and S-I width in the present study were 31.80 mm and 8.34 mm whereas for normal individuals they were 35.31 mm and 8.60 mm. The difference was statistically significant regarding anteroposterior length (*p* < 0.05). However, Shankar et al. reported no significant difference pertaining to both length and width of type 2 soft palate.

Type 3 (butt-like) soft palate was seen in 4 cases of stage II OSMF and 6 cases of stage III OSMF. The A-P length was 28.99 mm in the study group and 30.64 mm in normal individuals. The S-I width in the study and control group was 11.66 mm and 11.42 mm, respectively. There was a significant difference in A-P length. Even as there was a mild difference observed in S-I width, the difference was not statistically significant (*p* > 0.05). Considering the findings, a slight increase in the width and reduction in the length of soft palate were found. Similar findings were observed in the study conducted by Shankar et al. [13], while contradictory results were obtained according to Verma et al. [16].

Type 4 (straight line) variety of soft palate was seen in one case in each of stage I and II OSMF. The mean A-P length in study and control group was 28.98 mm and 35.15 mm, respectively. The S-I widths between the study group (9.80 mm) and control group (5.62 mm) were compared. Even though the length and width were less in the study group when compared to control group, the results were not statistically significant which may be due to small sample size.

Pépin et al. found the S-shape (type 5) which had hooked appearance of the soft palate in their study, with angulation of about 30° between distal part of uvula and longitudinal axis of soft palate. Therefore, the study concluded that hooked appearance in awake patients indicates a high risk for obstructive sleep apnea syndrome [18]. Kumar and Gopal [15] conducted a study in 100 normal individuals to evaluate the soft palate patterns and found only 2% of the cases showing S-shape (type 5) pattern of soft palate. Praveen et al. [14] reported 2.5% of the cases presented with this pattern. Amusingly, this type of soft palate was not observed in our study.

Type 6 (crook shaped) soft palate was seen in 5 cases of stage III OSMF. The mean A-P length was 30.89 mm whereas for normal individuals it was 31.9 mm and the mean S-I widths were 10.95 mm and 9.14 mm in normal individuals. There was a significant difference concerning the S-I width.

Stage I OSMF was seen in 7 cases where A-P length was 32.96 mm and S-I width was 8.97 mm and where the length is almost comparable with normal individuals, that is, 35.29 mm. In stage II OSMF, A-P length and S-I width were 32.70 mm and 10.00 mm and for stage III they were 29.98 mm and 11.18 mm, respectively. But the change is more pronounced in A-P length than in S-I width. These changes can be attributed to the fact that the length of the soft palate and uvula corresponds to the stage of the disease, that is, gradual shortening of soft palate and uvula with the progression in the staging of OSMF.

In our study, there was a gradual change in the dimensions and patterns of soft palate with advance in staging of OSMF and interestingly a negative correlation was observed in staging of OSMF and anteroposterior dimensions. That is, as the staging advances, the anteroposterior dimension decreases, but the superoinferior dimension increases along with staging. Type 1 and 2 variants of soft palate were predominantly seen in initial stages of OSMF, whereas type 3 and 6 variants were observed in advanced stages of OSMF. There is further scope for validating this field with a larger sample size.

## Figures and Tables

**Table 1:** Comparison of anteroposterior (A-P) length and superoinferior (S-I) width in the study and control group using Kruskal Wallis test.

Type of soft palate	Length of soft palate	Group	*N* (number) (%)	Mean	Standard deviation	*p* value
Type 1	A-P length	Study group	24 (52.2%)	34.00	2.552	0.001^*∗*^
		Control group	22 (47.8%)	36.82	1.107	
	S-I width	Study group	24	10.14	.629	0.007^*∗*^
		Control group	22	9.73	.400	

Type 2	A-P length	Study group	9 (34.6%)	31.80	.993	0.001^*∗*^
		Control group	17 (65.4%)	35.31	.893	
	S-I width	Study group	9	8.34	.770	0.062
		Control group	17	8.60	.357	

Type 3	A-P length	Study group	10 (66.7%)	28.99	1.382	0.020^*∗*^
		Control group	5 (33.3%)	30.64	.772	
	S-I width	Study group	10	11.66	.538	0.327
		Control group	5	11.42	.326	

Type 4	A-P length	Study group	2 (40.0%)	28.98	1.202	0.083
		Control group	3 (60.0%)	35.15	.507	
	S-I width	Study group	2	9.80	.778	0.083
		Control group	3	5.62	.195	

Type 6	A-P length	Study group	5 (62.5%)	30.89	1.269	0.230
		Control group	3 (37.5%)	31.90	1.294	
	S-I width	Study group	5	10.95	.325	0.025^*∗*^
		Control group	3	9.14	.259	

^*∗*^Statistically significant, *p* < 0.05.

**Table 2:** Mean distribution of anteroposterior (A-P) length and superoinferior (S-I) width in the study group and control group using Mann-Whitney *U* test.

Velar dimensions	Group	*N* (total number)	Mean	Standard deviation	*p* value
A-P length	Study group	50	32.09	2.834	0.001^*∗*^
	Control group	50	35.29	2.209	

S-I width	Study group	50	10.19	1.220	0.001^*∗*^
	Control group	50	9.23	1.288	

^*∗*^Statistically significant, *p* < 0.05.

**Table 3:** Cross tabulation for type of soft palate and staging of OSMF.

Type of soft palate	Stage I	Stage II	Stage III	Total
Type 1	2	19	3	24
	8.33%	79.16%	12.5%	100%

Type 2	4	5	0	9
	44.44%	55.56%	0.00%	100%

Type 3	0	4	6	9
	0.00%	44.44%	66.66%	100%

Type 4	1	1	0	2
	50%	50%	0.00%	100%

Type 6	0	0	5	5
	0.00%	0.00%	100%	100%

**Table 4:** The mean anteroposterior (A-P) length and superoinferior (S-I) width of soft palate patterns according to stagewise distribution of OSMF using Kruskal Wallis test.

Velar dimensions	OSMF staging	*N*	Mean	Standard deviation	*p* value
A-P length	Stage I	7	32.96	1.844	0.002^*∗*^
	Stage II	29	32.70	3.055	
	Stage III	14	29.98	1.385	
	Total	50	32.09	2.834	

S-I width	Stage I	7	8.97	1.001	0.001^*∗*^
	Stage II	29	10.00	1.152	
	Stage III	14	11.18	.592	
	Total	50	10.19	1.220	

^*∗*^Statistically significant, *p* < 0.05.

**Table 5:** Correlation of anteroposterior (A-P) length and superoinferior (S-I) width with the staging of OSMF using Spearman rank correlation.

	A-P length	S-I width
Staging of OSMF	−.461^*∗*^	.619^*∗*^
*p* value	0.001	0.000
*N* (total number)	50	50

^*∗*^Statistically significant, *p* < 0.05.
